# Cinematic Rendering of Bone SPECT/CT

**DOI:** 10.1097/RLU.0000000000006148

**Published:** 2025-11-18

**Authors:** Martin W. Huellner, Klaus Engel, Harald Essig, Bernd Stadlinger

**Affiliations:** * Department of Nuclear Medicine, University Hospital Zurich, University of Zurich, Zurich, Switzerland; † Siemens Healthineers AG, Erlangen, Germany; ‡ Clinic of Cranio-Maxillofacial and Oral Surgery, Center for Dental Medicine, University of Zurich, Zurich, Switzerland

**Keywords:** jaw, hyperplasia, tomography, emission-computed, single-photon, bone and bones, growth disorders, nuclear medicine, image processing, computer-assisted

## Abstract

A 14-year old boy with pronounced facial asymmetry was referred for [^99m^Tc]Tc-3,3-diphosphono-1,2-propanodicarboxylic acid (DPD) single-photon emission computed tomography / computed tomography (SPECT/CT) of the jaw. The initial clinical suspicion of causative fibrous dysplasia in the retromolar region was ruled out through both imaging and bone biopsy. Instead, [^99m^Tc]Tc-DPD-SPECT/CT confirmed the presence of hemimandibular hyperplasia. Cinematic rendering provided photorealistic images, effectively visualizing the asymmetry of the mandible and teeth, and the underlying increased condylar bone turnover.

**FIGURE 1:**
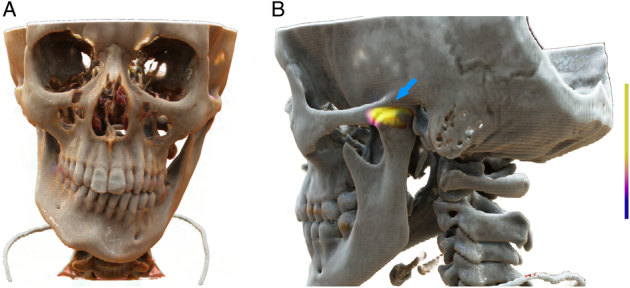
The image shows a cinematically rendered standard digital [^99m^Tc]Tc-DPD-SPECT/CT image (NM SPECT/CT 870, GE HealthCare, Waukesha; CT image: tube voltage 120 kV, adaptive tube current modulation, reconstructed slice thickness 0.625 mm; SPECT image: 650 MBq of [^99m^Tc]Tc-DPD, step time 0:30 minutes, step angle 3 degrees, total angular range 360 degrees, reconstructed iteratively) in a patient with pronounced facial asymmetry (**A**) caused by left-sided hemimandibular hyperplasia with increased bone turnover in the left-sided mandibular condyle (**B**, arrow). The mandible is likely the bone affected by the widest variety of diseases, many of which can be effectively evaluated using various nuclear medicine imaging techniques with different tracers.^1–14^ Hemimandibular hyperplasia, or in its early form, condylar hyperplasia, is a growth disorder of the mandible, typically encountered in children and adolescents. The disease is characterized by often unilateral or asymmetrically increased bone turnover in the mandibular condyle. This is associated with excessive growth of one hemimandible, with clinical consequences ranging from malocclusion and misaligned teeth to pronounced facial asymmetry.^15^ [^99m^Tc]Tc-DPD and other bone-seeking radiotracers are established scintigraphic techniques for the assessment of a variety of bone disorders, including the search for pathologic condylar growth activity in hemimandibular hyperplasia.^16^ Cinematic rendering represents a novel technology that transforms how radiologists and other medical specialists such as surgeons perceive anatomical structures and pathological conditions. By merging principles of radiology and cinematography, cinematic rendering surpasses traditional rendering methods, producing lifelike representations that bridge the gap between medical images and reality.^17,18^ This post-processing technique relies on simulating visible light interaction with standard fused radiological images to generate photorealistic 3-dimensional representations, thereby improving spatial orientation.^19^ Although its primary application has historically focused on CT imaging,^19–23^ with occasional use in PET/CT^19^ and PET/MR,^18^ its implementation in SPECT/CT has not yet been reported. The cinematically rendered image presented here was retrospectively reconstructed from a standard clinical image data set. These photorealistic images have the potential to assist radiologists in diagnostics and surgeons in preoperative planning by providing critical anatomic landmarks, enhancing visualization of subsurface structures, and complementing endoscopic imaging. In particular, cinematic rendering has proven useful in anatomical regions with intricate detail and numerous small structures, where 3-dimensional orientation is crucial—such as the head and neck.^22,24–26^
